# Electrochemical Reduction of Coumarins at a Film-Modified Electrode and Determination of Their Levels in Essential Oils and Traditional Chinese Herbal Medicines

**DOI:** 10.3390/molecules14093538

**Published:** 2009-09-11

**Authors:** Lai-Hao Wang, Hsiu-Hua Liu

**Affiliations:** Department of Applied Chemistry, Chia Nan University of Pharmacy and Science, 60 Erh-Jen Road, Section 1, Jen Te, Tainan 71743, Taiwan; E-mail: appys31@yahoo.com.tw (H-H. L.)

**Keywords:** coumarins, essential oils and medicinal products, mercury-film electrode

## Abstract

The electrochemical reduction of coumarins on glassy carbon and electrodeposited metal electrodes was investigated in a Britton-Robinson buffer (pH 1.87-11.98). The effects of various factors, such as the deposition material, time, and concentration of mercury, on the precision of the analysis were explored. The possible reaction mechanism of the reduction process with regards to scan rates, peak potentials, and currents is discussed. Electroreduction was used to quantitatively determine the levels of coumarins in some essential oils and traditional Chinese herbal medicines. A comparison with high performance liquid chromatography analysis results shows good agreement.

## 1. Introduction

Coumarins, with a 2*H*-1-benzopyran-2-one nucleus, and furocoumarins (psoralens, 7H-furo[3,2-g[1]benzopyran-7-one]) are biologically active compounds with effective anti-convulsant, anti-tumoral, anti-inflammatory, and anti-viral properties [1,2,3,4,5,6,7]. Additionally, coumarins have been widely used as flavoring compounds because of their sweet and aromatic odor [8]. Coumarin dyes are organic materials with high solar-to-energy transfer-efficiency in dye-sensitized solar cells (DSC) [9]. Therefore, overall coumarins are considered important natural compounds.

Various spectrophotometric [10,11,12,13], liquid chromatography-mass spectrometric (LC-MS) [14,15,16,17,18,19,20,21,22,23,24], and gas chromatography-mass spectrometric (GC-MS) [25,26,27] methods have been used to determine coumarin levels in plants and foods. However, most of these methods involve only the extraction and identification of chemical constituents in natural plants. There are a few reports in the literature [28,29] on the determination of urinary metabolites after ingesting medicinal plants, but there are only a few published reports [30,31,32,33] on the behavior of coumarin derivatives. Furthermore, there is only one published report [34] concerning the use of using electrochemical oxidation to determine coumarin levels in plants.

In this work, we electrochemically reduced coumarins using a bare glassy carbon electrode (GCE) and mercury (II)-nitrate-[Hg(NO_3_)_2_] and lead-acetate-[Pb(CH_3_COO)_2_] modified-GCEs, respectively. We investigated coumarin levels using direct current as well as cyclic and differential pulse voltammetry (DPV). The optimum experimental conditions for determining coumarin levels in some essential oils and traditional Chinese herbal medicines are also described in this paper.

## 2. Results and Discussion

### 2.1. Choice of analytical method

**Scheme 1:**
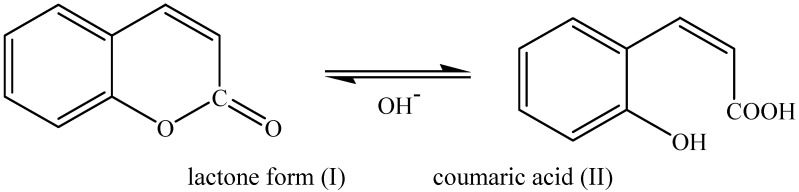
Coumarin: Both forms depend on pH.

The polarographic behavior of coumarins indicates that at a pH > 11.2, they exist entirely in the lactone form (I), and at a pH < 6.8 entirely as coumaric acid (II) (Scheme 1); both forms are present when the pH is between 6.8 and 11.2 [35].

The reduction of coumarin in lithium perchlorate was studied on a GCE, thin-film mercury deposited on a GCE (Hg/GCE), and thin-film lead deposited on a GCE (Pb/GCE). Peak potentials were −1.370 V (GCE), −1.360 V (Pb/GCE), and −1.356 V (Hg/GCE); peak currents were 11.818 μA (GCE), 15.457 μA (Pb/GCE), and 20.537 μA (Hg/GCE), which indicated that Hg/GCE performed best. Because the thin-film mercury was more sensitive than the dropping mercury electrode [36], the Hg/GCE was used to determine the levels of coumarins in the essential oils and traditional Chinese herbal medicines.

The deposition conditions primarily affect the specific surface area of the metal catalyst. We investigated the optimum conditions for electrochemically depositing metal onto the GCE. We investigated the effect of the thickness of the metal layer by coating the GCE with deposition solution at 2, 4, 6, and 8 min, respectively. Mercury film was electrochemically deposited for 4 min onto a GCE in a 0.1-M acetate buffer aqueous solution of 2.06 × 10^−3^ M of Hg (NO_3_)_2_ at a potential between 0.0 and +1.2 V (*vs.* Ag/AgCl) at 10 mV/s. These values were used because the slope and the linear concentration range of the calibration graph were the largest using them than using other values.

**Figure 2:**
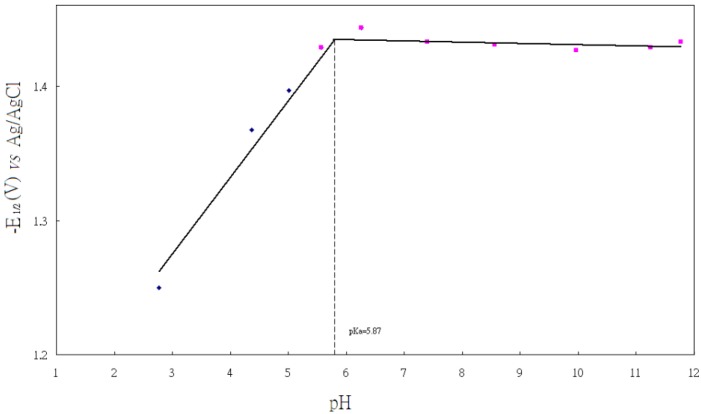
Plots of –E_1/2_ *vs.* pH for coumarin. Concentration: 6.22 × 10^−4^ M.

The effect of pH on the E_1/2_ (half-wave potential) value of coumarin was studied over the pH range of 2-12. The average values of dE_1/2_/dpH of coumarin in acidic and basic solutions were 0.057 and 0.0018 V/pH, respectively. The result conformed to the relationship shown in the following equation:

E_1/2_ = constant + RT/αn_a_F ln [H^+^]/K + [H^+^] (1)

and indicated that, at a low pH, the half-wave potentials rose as the pH increased. However, the half-wave potentials became constant at a pH > 8. The E_1/2_ values and pH values were keyed into the computer, and then the acid dissociation constant (p*K*_a_) value was determined using simple regression. The intersection (breakpoint) of the two linear parts occurred when the p*K*_a_ = 5.87 (Figure 2).

We then did comparative tests of supporting electrolytes — Britton-Robinson (BR) buffer (pH 1.87-12.05) and aqueous solutions containing phosphate buffer (pH 6.52), acetate buffer (pH 4.04), and 0.1 M lithium perchlorate (pH 6.02). Differential pulse voltammograms of coumarin in BR buffered solution in the pH range of 5.01-10.01 at the Hg/GCE showed two well-defined reductions at −0.733 V and −1.423 V (Table 1).

**Figure 3:**
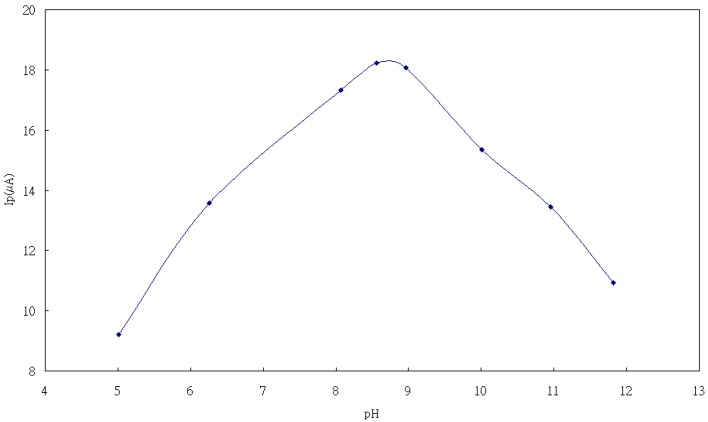
The effect of pH on the response current of 6.22 × 10^−4^ M coumarin in Britton-Robinson (BR) buffer at a thin-film mercury modified GCE (Hg/GCE); differential pulse voltammetry (DPV) scan rate: 10 mV/s.

It seems reasonable that there is a 1-electron reduction to a dimer [37]. This pH agrees with that (9.96-10.96) at which the peak height markedly decreases. Hence, we concluded (i) that the lactone (I), which is, based on chemical evidence, the stable form in acid media, is reducible over the range of potentials studied; (ii) that the acid (II) is not reducible over that range; and (iii) that (I) is present to a negligible extent above pH 11.77 and (II) to a negligible extent below pH 4.86. Above pH 10, the current decreases, because the lactone ring is opened to a non-reducible anion. Below pH 4, there was no peak because of the overlying hydrogen discharge. The plot of Ip *vs.* pH (Figure 3) and the maximum peak current were obtained at pH < 9. For analytical purposes, the best supporting electrolytes for determining coumarins are BR buffers (pH 8.07-8.96) because they are more sensitive than others.

### 2.2. The Hg/GCE catalyzed the reduction of coumarin

**Figure 4:**
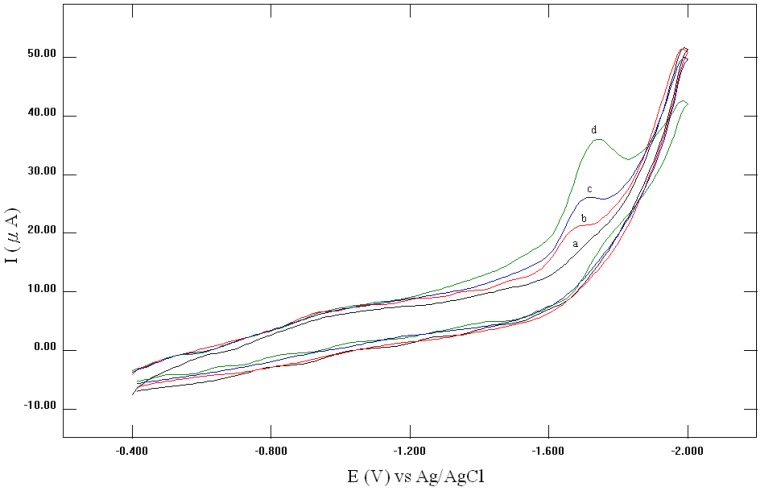
Cyclic voltammograms of coumarin in Britton-Robinson (BR) buffer (pH 5.83) at a thin-film mercury-modified GCE (Hg/GCE). Concentrations: (a) 2.723 × 10^−4^ M; (b) 5.446 × 10^−4^ M; (c) 1.089 × 10^−4^ M; (d) 2.178 × 10^−4^ M; scan rate: 12.5 mV/s.

Cyclic voltammograms were recorded at different concentrations as shown in Figure 4. The I_p_ is proportional to concentration, the regression equation was:

y = 14.3 + 11.8 × (correlation coefficient: r = 0.9981)(2)

**Figure 5:**
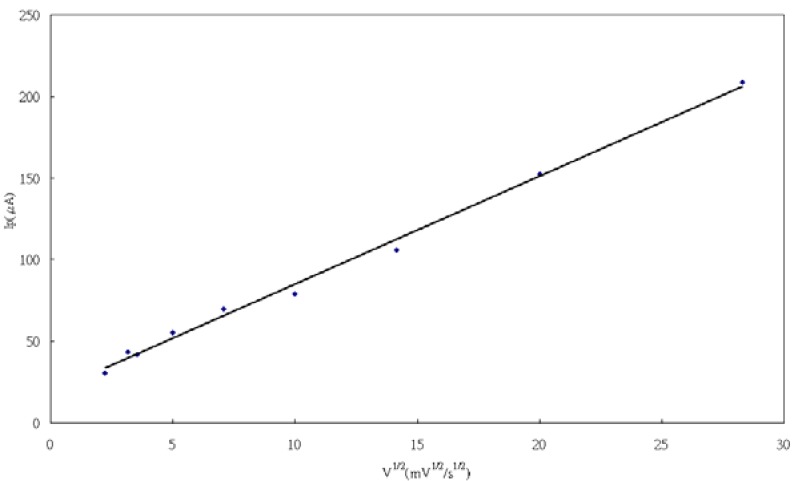

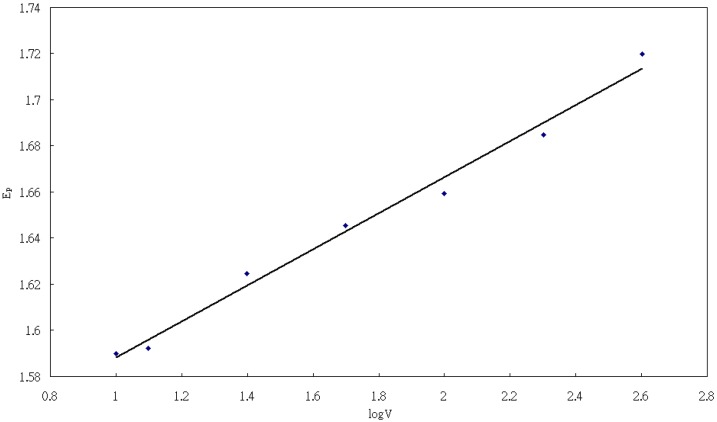
(A) Magnitude of peak current (Ip) as a function of the square of the scan rate for coumarin reduction. Concentration: 3.73 × 10^−3^ M. (B) Real potentials of coumarin reduction as a function of the logarithm of the scan rate. Concentration: 3.73 × 10^−3^ M.

The linearity between the peak current (I_p_) and the square root of the scan rate *v^1/2^* (Figure 5A) good, and it was characteristic of a diffusion-controlled process. The relationship between peak potential and the logarithm of the scan rate (Figure 5B) can be used to roughly estimate the number of electrons involved in the catalytic reduction. For a totally irreversible electrochemical reduction, the peak current in cyclic voltammograms can be expressed as:

I_p_ = (2.99 × 10^5^) n(αn_a_)^1/2A C^_0_D_0_^1/2^*v*^½^ (3)

where I_p_ is the peak current, n is the number of electrons involved in the reduction, αn_a_ is a parameter reflecting the irreversibility of the reduction, A is the area of the electrode (cm^2^), C_0_ is the concentration of substrate, *v* is the potential scan rate, and D_0_ is the diffusion coefficient of the substrate. The value of n(αn_a_) can be calculated from the slope of the line in Figure 5A. On the other hand, the peak potential in cyclic voltammograms can be expressed as a function of the logarithm of the scan rate:

E _p_ = k + (0.03/αn_a_) log *v.* (4)

Therefore the number of electrons (n) involved in the reduction process can be obtained from data shown in Figures 5A and B; a value of n = 0.94 electrons was obtained.

**Scheme 2:**
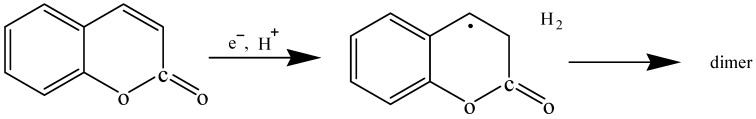
Possible mechanism.

Therefore, a possible mechanism is given below in Scheme 2:

### 2.3. Reproducibility and accuracy

Recovery tests were done on the essential oils and traditional Chinese herbal medicines to evaluate the reproducibility and accuracy of the proposed differential pulse voltammetry (DPV) method. Essential oils and traditional Chinese herbal medicines were spiked with the amounts reported in Table 2 and subjected to the whole procedure. Excellent recoveries (recoveries ranging from 97 ± 4.0% to 99 ± 2.7%) and precision were obtained (Table 2). The calibration plots obtained by plotting the peak current against the level of coumarins show good linearity over the range of 2-60 mg/L. For coumarin, the regression equation was:

y = 5.464 + 0.1853 × (correlation coefficient: r = 0.9990),(5)

and for psoralen:

y = 5.25 + 0.2351 × (r = 0.9989).(6)

The relative standard deviation values were between 2.4% and 4.5%.

### 2.4. Application to essential oils and traditional chinese herbal medicines

**Figure 6:**
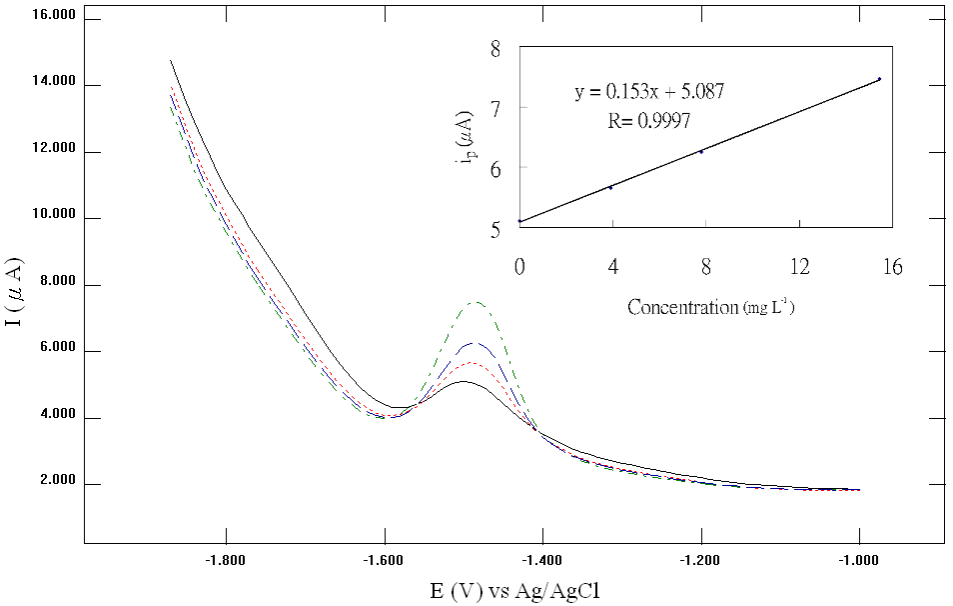
DPV for 8-MOP from *Angelicae dahuricae* at an Hg/GCE using a standard addition procedure. The peak current values were (i) solid line: 5.114 μA at −1.490 V with 0 mg L^−^^1^ of 8-MOP; (ii) dashed line: 5.659 μA at −1.488 V with 4.0 mg L^−^^1^ of 8-MOP; (iii) dotted line: 6.256 μA at −1.482 V with 8.0 mg L^−^^1^ of 8-MOP; and (iv) dashed/dotted line: 7.461 μA at −1.482 V with 16 mg L^−^^1^ of 8-MOP. Scan rate: 10 mV/s; pulse height: 50 mV.

The proposed DPV method was used to determine the levels of coumarins in essential oils and traditional Chinese herbal medicines. For *Angelicae dahuricae,* a traditional Chinese herbal medicine, using standard solutions of 8-methoxypsoralen (8-MOP), the peak height of the wave was at −1.490 V (Figure 6). Hence, in this study, determining coumarin levels in commercial essential oils and traditional Chinese herbal medicines used a standard addition procedure. The analytical results (Table 3) agreed with those obtained using high-performance liquid chromatography (HPLC).

## 3. Experimental

### 3.1. Apparatus

All electrochemical experiments were done using an EG&G Princeton Applied Research Model 394 Polarographic Analyzer (Princeton, NJ, USA). We used a three-electrode system consisting of a working electrode (Hg(NO_3_)_2_/GCE, Pb(CH_3_COO)_2_/GCE, and GCE), a platinum counter, and an Ag/AgCl reference electrode. HPLC was done with a Hitachi model L-7100 pump and model 7125 injector equipped with 20-μL sample loop and a model L-7455 photodiode array detector. Chromatograms were acquired and peak areas calculated using a D-7000 chromatogram data integrator.

### 3.2. Reagents and materials

Coumarin and 5-methoxypsoralen (bergapten, 5-MOP) were purchased from Sigma-Aldrich Chem. Co. (St Louis, MO, USA). Psoralen and 8-methoxypsoralen (xanthotoxin, 8-MOP) were purchased from the National Institute for the Control of Pharmaceutical and Biological Products (Beijing, China) and Tokyo Chemical Industry Co., Ltd (Japan), respectively. All other chemicals were analytical reagent grade. Samples of essential oils and medicinal products were bought from a number of retail outlets in southern Taiwan. The BR buffer solutions (pH 1.87-12.05) were prepared by mixing 0.5 M phosphoric acid, 0.5 M boric acid, 0.5 M acetic acid, and 0.2 N sodium hydroxide solutions; pH was checked using a pH meter.

### 3.3. Procedures

#### 3.3.1. Voltammetric measurements

The modified Hg/GCE and Pb/GCE were produced using the following method. A GCE was electrolytically plated with mercury or lead ions from 10 ml of acetate buffer (pH 4.13) that was 5.0 × 10^−4^ to 2.0 × 10^−3^ M mercury (ΙІ) or lead (II). Plating time was 2-8 min; potential was scanned from −1.0 to 0.0 V. Sample DC polarography, cyclic and linear sweep, and differential pulse voltammograms were taken of coumarins in BR buffer solutions at Pb- and Hg-modified GCEs.

#### 3.3.2. Sample preparation

Taking into account the content of coumarins or psoralens in the sample, about 0.5-1.0 g of the latter was accurately weighed, mixed with methanol and deionized water (1:1, v*/v*), and then stirred and refluxed at 70 °C in a water bath for 1 h. The extract was separated from samples using centrifugation at 6,000 rpm for 30 min. The supernatant volume was extracted with hexane-ethyl acetate (3:1, *v/v*) and evaporated to dryness with a stream of nitrogen. The above procedure was repeated three times. The supernatant was transferred into a 10-mL calibrated flask and made up with methanol.

#### 3.3.3. Determining coumarin levels using DPV

To obtain calibration plots for the coumarins, 10 mL of supporting electrolytes were pipetted into a voltammetric cell and deaerated with nitrogen for 4 min before voltammetric measurement. Using a micropipette, aliquots of coumarin solutions (1,000 mg/L^−1^) were added and left to deaerate for 2 min. Voltammograms were then taken. Quantitative analyses were done in the differential pulse mode. The potential was set from −0.4 to −2.0 V *vs.* Ag/AgCl. The pulse height was 50 mV and the scan rate was 10 mVs^−1^ with a drop time of 1.0 s. For sample solution analysis, 0.1 mL of the solution was pipetted into a 10-mL calibrated flask and diluted to volume with BR buffer. The solution was analyzed using DPV under the same conditions as for the calibration plot.

#### 3.3.4. Determining coumarin levels using liquid chromatography

A stock standard solution was prepared by dissolving 10 mg of coumarins and psoralens in 10 mL of methanol, respectively. Working standard solutions were prepared from a stock standard solution, in the range 10-80 mg/L^−1^, in methanol. Reversed phase (RP)-HPLC was done on a Phenomenex Luna C_18_ (5u, 250 × 4.6 mm) column with eluted methanol-water (45:55, *v/v*) as the mobile phase at 1 mL/min^−1^. Detecting coumarin levels after separation on the C_18_ column was done using a photodiode array detector. Using an injection value, 20 μL of the prepared sample solution and standard solution was chromatographed under the operating conditions described above. Quantitation was based on the peak area of the sample.

## 4. Conclusions

The electroreduction of coumarins was more sensitive on thin-film mercury electrodes than on glassy carbon electrodes. A thin layer of metal deposited on the cathode surface is sufficient to make the cathode effective, and the thickness of the deposit can be easily adjusted. Electrodeposited metals also offer other advantages compared with cast electrodes. The DPV method described in this paper is simple and can be used for directly monitoring the coumarin levels in essential oils and traditional Chinese herbal medicines.

## Figures and Tables

**Figure 1:**
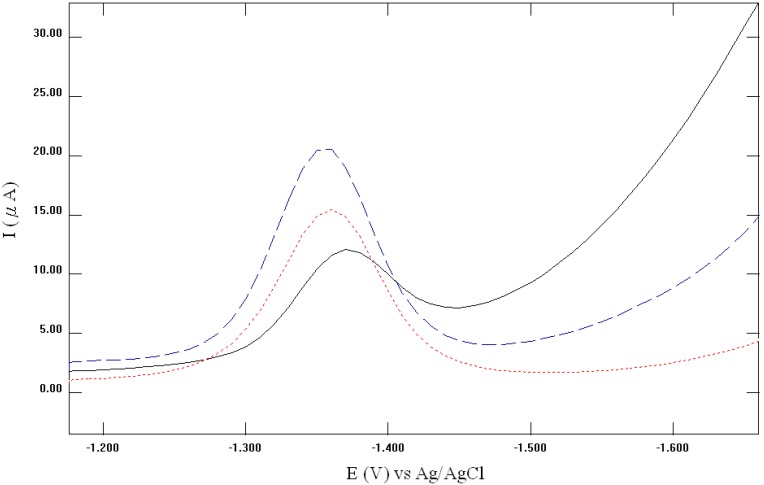
Differential pulse voltammograms of coumarin (6.22 × 10^−4^ M) in lithium perchlorate: solid line (−) at GCE; dotted line (…) at lead-modified GCE (Pb/GCE); dashed line (---) at mercury-modified GCE (Hg/GCE); scan rate: 10 mV; pulse height: 10 mV.

**Table 1:** The effect of pH and supporting electrolytes on the differential pulse voltammetric peak potential and peak current of coumarin at mercury-modified glassy carbon electrode (Hg/GCE).

Medium	pH	E_p_(V)	i_p_(μA)
BR buffer	5.01	−0.733 −1.423	1.086 9.206
BR buffer	6.82	−0.785 −1.470	2.81 913.673
BR buffer	7.46	−0.768 −1.463	4.730 17.239
BR buffer	8.56	−0.736 −1.429	2.258 18.217
BR buffer	9.46	−0.713 −1.399	2.673 17.796
BR buffer	9.96	−0.756 −1.421	2.548 14.539
BR buffer	10.01	−0.797 −1.412	2.321 15.353
Phosphate	6.52	--^a^ -1.428	--^a^ 20.889
Acetate buffer	4.04	--^a^ −1.348	--^a^ 11.664
LiClO_4_	6.02	--^a^ −1.413	--^a^ 13.513

BR, Britton-Robinson; ^a^ Not determined.

**Table 2:** Recoveries of coumarins in essential oil and traditional Chinese herbal medicines using differential pulse voltammetry (DPV).

Samples	Coumarin			Psoralen		
	Added	Found	Recovery	Added	Found	Recovery
	(mg/L)	(mg/L)	(%)	(mg/L)	(mg/L)	(%)
Lemon essential oil	16.0	14.8	99 ± 2.7	---	---	---
*Psoralea corylifolia*	---	---	---	8.00	7.92	99 ± 3.5
*Cnidium monnieri*	---	---	---	8.00	7.76	97 ± 4.0

**Table 3:** Analytical results of determining coumarin levels in commercial pharmaceutical preparations using differential pulse voltammetry (DPV) and liquid chromatography with ultraviolet detection (LC-UV). ^a^

	Concentration (%, w/w), N = 5^b^							
	Coumarin		Psoralen		5-MOP		8-MOP	
Samples	DPV	LC-UV	DPV	LC-UV	DPV	LC-UV	DPV	LC-UV
Bergamot essential oil	---^c^	---^c^	---	---	0.203 (5.1%)	0.206 (4.6%)	---	---
Lime essential oil	0.034 (1.9%)	0.031 (1.6%)	---	---	---	---	---	---
Lemon essential oil	0.202 (5.2%)	0.191 (2.4%)	---	---	0.022 (4.5%)	0.020 (3.9%)	---	---
*Psoralea corylifolia*	---	---	0.148 (4.3%)	0.143 (3.3%)	---	---	---	---
*Angelicae dahuricae*	---	---	---	---	0.010 (2.3%)	0.009 (1.1%)	0.005 (0.31%)	0.0045 (3.3%)
*Cnidium monnieri*	---	---	0.004 (1.4%)	0.003 (2.5%)	0.019 (5.1%)	0.020 (0.3%)	0.0022 (4.5%)	0.0020 (4.3%)

^a^ Number of determinations. ^b^ Values in parentheses are the relative standard deviation. ^c^ Not determined.
